# Universal Health Coverage as a Distinct Sustainable Development Goals Target: Dispelling Doubts and Underlining Implications

**DOI:** 10.3389/fpubh.2015.00238

**Published:** 2015-10-15

**Authors:** Mahip Acharya

**Affiliations:** ^1^Independent Researcher, Kathmandu, Nepal

**Keywords:** sustainable development goals, universal health coverage, desirable health outcome, health equity, public health measures

United Nations General Assembly is preparing proposal for Sustainable Development Goals (SDGs), and it has recently made explicit its working group proposal. Health-related SDG (SDG3) focus on health, envisioning healthy lives for people at all ages. Targets, such as reducing the global maternal mortality ratio to <70 per 100,000 live births by 2030, ending preventable deaths of newborns and under-five children by 2030, and halving global deaths and injuries from road traffic accidents by 2020, among others, have been set to attain the goal. One among them is to achieve universal health coverage (UHC), ensuring financial health protection and accessibility to essential health services for all (1).

Universal health coverage has permeated the health discourse and is garnering worldwide attention. Despite that, it is an unusual target, for it has traditionally been regarded as a means for – and not a component of – better health outcomes. Millenium development goals (MDGs), the predecessor to SDGs, included health-related targets along the lines of reducing under-five mortality, maternal mortality, and halting the spread of HIV/AIDS, malaria, and other diseases. Inclusion of UHC in SDG3 has been taken with a pinch of salt, and doubts have been raised whether this undermines public health measures and health equity (2). Notwithstanding some practical issues, setting UHC as a SDG target has positive implications for health equity and overall health status.

First, UHC is a desirable outcome of a health system, even if it does not inevitably lead to improvement in other health measures. Providing accessibility to essential health services and ensuring financial risk protection are in themselves good features of a health system and are worth striving for (3, 4). Further, evidence has shown that UHC, in fact, leads to improved population health (5). UHC, thus, can as much be a means for better health as a component of it, and it is prudent to select it as a SDG target.

Second, UHC when attained has a positive bearing on health equity (6). But, this is not as simple because complete attainment of UHC requires many years of persistent efforts. Additionally, there are different paths for it – as is shown by diverse political, economic, and policy measures adopted by different countries. Concerns have been brought up as to the pursuit of UHC contributing to inequity in health outcomes – to be precise, these concerns pertain to the early stages in the transition to UHC, as it has been shown that poor and disadvantaged groups do not immediately benefit (7). These doubts call into question the fascination with UHC and its being considered as a SDG target. But, it has been observed that population-wide health measures, initially, tend to escalate health inequity, even when UHC is not the ultimate goal (8). Additionally, the quest for UHC need not be anti-poor, for there are counterexamples of Mexico and Brazil, where the progress toward UHC was pro-poor (7). Further, targeting the poor as a complementary approach to UHC has proved beneficial in reducing the health gap between rich and poor, as observed in Indonesia (9). Approaches, it seems obvious, a country take for UHC, and not the progress toward UHC in general, are the decisive factor for health equity. Moreover, monitoring and evaluation forms an important and often an integral part of UHC and is helpful in bringing out health reality on the ground (3). Monitoring progress toward UHC by disaggregating data on the basis of socioeconomic characteristics (such as wealth quintile) and demographic variation (such as urban-rural residence) helps uncover inequitable health distribution (3). Provided there is strong commitment, such information elicits policy responses that are meant to tackle this inequity, as shown by experiences of Mexico, Brazil, and Indonesia. So, setting UHC as a SDG target is in line with health equity. In addition, SDG3 targets, such as reducing global mortality rate to <70 per 100,000 live births and reducing pre-mature mortality from non-communicable diseases by one third, could still be achieved with substantial inequality among different socioeconomic and demographic groups. In this regard, UHC, when achieved, could be invaluable in reducing the gap, as it encourages the utilization of health services, including the public health ones, among the entire population, poor and marginalized groups included. Further, non-health SDGs, such as ending poverty, ending hunger, and ensuring inclusive and equitable quality education, have a positive influence on health and disproportionately so on the health of poor (10). Conversely, better health is known to positively bear on ending poverty and ensuring education (11). Non-health SDGs in conjunction with UHC, if and when achieved, can thus ascertain better health and health equity.

Third, UHC is compatible with public health measures. UHC is often criticized for its alleged emphasis on curative services with disregard for preventive and promotive ones. Besides, SDG3 targets (such as ending the epidemics of AIDS, tuberculosis, and malaria, and ending preventable deaths of newborn and under-five children, to name a few) call for population-level health measures, and reaching these targets and achieving UHC, it is claimed, do not usually go hand in hand. Excess emphasis on curative services does divert attention and resources from all-encompassing, low cost, and effective public health services. But, UHC does not automatically mean covering curative health services, more so for low- and middle-income countries. In low-income countries (as well as in middle-income ones), measures such as vaccination, malaria and tuberculosis control (largely through prevention), and eradication of malnutrition, among others, are considered as the essential services, which need to be universally covered (12). Although most of these are private goods with quasi-public good characteristics, they can loosely be referred to as public health programs (13). Improving and expanding primary health care has been recommended as a better path for achieving and sustaining UHC (14). Further, WHO’s world health report 2010, which exclusively foregrounds UHC, categorically stated health promotive and preventive services as essentials of UHC (4). Although reports and recommendations on achieving UHC do not necessarily translate into policy formation and implementation, empirical evidences from low- and middle-income countries, such as Rwanda (15), Costa Rica (16), Kyrgyzstan (17), Thailand (18), and Moldova (19), show that this can be done. Countries which are working toward UHC, such as Ethiopia, are investing on community health workers (20), who predominantly provide promotive and preventive services. However, in high- and upper middle-income countries with UHC, shift toward curative health services in coverage package is observed. Australia is the recent one, where universally covered service package is shifting away from primary healthcare (21). This trend is partly because of the rise in elderly population with chronic diseases and greater prevalence of non-communicable diseases, public health measures for which are complex and go as far as behavior modification. SDG3 targets, such as strengthening implementation of Framework Convention on Tobacco Control and reducing the number of deaths and illness from hazardous chemicals and pollution, which necessitate actions that reach beyond health sector, are framed to tackle this growing burden of non-communicable diseases. UHC is a product of political, social, and economic systems, where allocation and distribution of health resources lie at its heart (22). As such, it is not averse to public health measures. Rather, there is a trade-off between different measures, the orientation of which is predicated on socioeconomic and political features of each country. In this regard, allocating a fixed share of UHC budget to health promotion and prevention activities and monitoring intersectoral effects on health could be instrumental to not letting health preventive and promotive measures dwindle, as suggested (2). Further, UHC as a SDG3 target, which primarily calls for health financing reforms, and other SDG3 targets which call for environmental and legal reforms are not contradictory; instead, they are complementary to one another. Time is ripe for a global march toward UHC, and, as is argued here, it does not negate, rather complements, other health measures.

The whole issue can be summarized in Table 1.

**Table 1:** **Summary**.

Arguments opposing UHC as a SDG target	Arguments supporting UHC as a SDG target
There are no abundant empirical evidences to ascertain that UHC leads to better population health	UHC is in itself a component of better population health
	Although we cannot claim that UHC invariably leads to better health, evidence, however limited, shows that it improves certain health indicators
Early stages in the progress toward UHC aggravate health inequity, for rich are the ones who benefit disproportionately	Health programs implemented such that they initially reach out to the poor have shown to contribute to health equity. Country studies of Mexico, Brazil, and Indonesia have observed this. Further, it is observed that public health measures, when introduced first, worsen health inequity, even when attaining UHC is not the target
Population health measures and attaining UHC fall under different SDG targets. This shows that UHC is predominantly concerned with curative health services	UHC is about assuring that all the people have access to health services. SDG requiring population-wide health interventions, such as reducing global mortality rate to <70 per 100,000 live births, could still be achieved with a segment of population not utilizing those services. UHC ascertains that those measures, and the benefits in health resulting from them, reach people of all socioeconomic status
Experiences of Thailand and, recently, Australia show that UHC inevitably favors curative services	Many low- and middle-income countries which have achieved UHC have done so riding on their population health and primary care programs. Further, allotting a fixed portion of UHC budget to preventive and promotive measures helps check the slide toward curative services. Finally, environmental and legal reforms complement health financing reforms (meant to achieve UHC)

## Conflict of Interest Statement

The author declares that the research was conducted in the absence of any commercial or financial relationships that could be construed as a potential conflict of interest.
